# Synthesis and characterization of lead-based metal–organic framework nano-needles for effective water splitting application

**DOI:** 10.1038/s41598-023-39697-z

**Published:** 2023-08-02

**Authors:** Ayman S. Eliwa, Mahmoud A. Hefnawy, Shymaa S. Medany, Reem G. Deghadi, Wafaa M. Hosny, Gehad G. Mohamed

**Affiliations:** 1 Chemistry Department, Faculty of Science, Cairo University, Giza, Egypt; 2 Nanoscience Department, Basic and Applied Sciences Institute, Egypt-Japan University of Science and Technology, Alexandria, Egypt

**Keywords:** Chemistry, Catalysis, Electrochemistry, Energy, Environmental chemistry, Green chemistry, Inorganic chemistry, Materials chemistry, Physical chemistry

## Abstract

Metal organic frameworks (MOFs) are a class of porous materials characterized by robust linkages between organic ligands and metal ions. Metal–organic frameworks (MOFs) exhibit significant characteristics such as high porosity, extensive surface area, and exceptional chemical stability, provided the constituent components are meticulously selected. A metal–organic framework (MOF) containing lead and ligands derived from 4-aminobenzoic acid and 2-carboxybenzaldehyde has been synthesized using the sonochemical methodology. The crystals produced were subjected to various analytical techniques such as Fourier-transform infrared spectroscopy (FT-IR), Powder X-ray diffraction (PXRD), scanning electron microscopy (SEM), energy dispersive X-ray (EDX), Brunauer–Emmett–Teller (BET), and thermal analysis. The BET analysis yielded results indicating a surface area was found to be 1304.27 m^2^ g^−1^. The total pore volume was estimated as 2.13 cm^3^ g^−1^ with an average pore size of 4.61 nm., rendering them highly advantageous for a diverse range of practical applications. The activity of the modified Pb-MOF electrode was employed toward water-splitting applications. The electrode reached the current density of 50 mA cm^−2^ at an overpotential of − 0.6 V (vs. RHE) for hydrogen evolution, and 50 mA cm^−2^ at an overpotential of 1.7 V (vs. RHE) for oxygen evolution.

## Introduction

The hydrogen evolution reaction (HER) is a process that produces hydrogen gas from water by applying an electric current. Hydrogen is a clean and renewable energy carrier that can be used for various applications, such as fuel cells, power generation, and chemical synthesis^1–7^. HER reduces the dependence on fossil fuels, which are the main sources of greenhouse gas emissions and air pollution^8^. By using water as a raw material, HER avoids the extraction and transportation of fossil fuels, which have negative effects on ecosystems and human health also, HER enables the integration of renewable energy sources, such as solar and wind, into the energy system^9^. Renewable energy sources are intermittent and variable, which poses challenges for grid stability and storage. By converting excess renewable electricity into hydrogen^10^. HER can balance the supply and demand of electricity and store energy for later use. In addition, HER supports the development of a circular economy, it can use wastewater or seawater as sources of water, thus reducing freshwater consumption and treating wastewater. Moreover, HER can use carbon dioxide as a co-reactant to produce synthetic fuels or chemicals, thus mitigating carbon emissions and creating value-added products^11,12^. Therefore, HER is a promising technology that can contribute to the transition to a low-carbon and sustainable society^13^.

The electrocatalytic process is considered an essential technique that is widely used in various applications like sensors, fuel cells, solar cells, and water splitting applications^14–19^. One of the promising strategies for producing clean hydrogen fuel is electrocatalytic hydrogen evolution (EHE), which involves splitting water molecules into hydrogen and oxygen using an electric current. However, EHE requires efficient and stable catalysts that can facilitate the reaction at low overpotentials and high current densities^20–23^. The considerable interest in metal–organic frameworks (MOFs) stems from their exceptional characteristics, including a large surface area, tunable pore size, precise metal positioning, and well-organized crystalline structure, as reported in literature^24,25^. MOF materials are recognized as electrochemically active catalysts that are extensively employed in electrochemical applications such as fuel cells, Li-batteries^26–28^, supercapacitors^29–31^, and water splitting^32,33^. This is in accordance with previous studies^34–36^. The utilization of MOF as a substrate for urea electrooxidation has been reported to be effective in urea removal. This is attributed to the substrate's extensive surface area, abundance of adsorption sites, proficient charge transfer capacity, and notable crystallinity, as documented in previous studies^37–40^.

The oxygen evolution reaction (OER) is a crucial process in various devices such as rechargeable metal-air batteries, water electrolysis systems, and solar fuel devices, as it acts as the limiting factor due to its excessively high overpotentials.

Electrocatalysis for the oxygen evolution reaction (OER) holds significant importance within the realm of advanced technologies, as it serves as a pivotal factor in enhancing the efficiency of gas evolution. Consequently, a multitude of novel electrocatalysts have been devised to further augment this process. Considerable resources have been dedicated to the pursuit of efficient electrocatalysts, prompting the development of novel methodologies for studying material properties and the underlying mechanisms of the oxygen evolution reaction (OER)^41^. Recently, knowledge not only serves as the basis for understanding the functioning of the OER mechanism, but also highlights the essential factors that contribute to the efficacy of an electrocatalyst, as evidenced by numerous research investigations^42,43^.

The optimization of the hydrogen evolution reaction (HER) and oxygen evolution (OER) is deemed crucial for the generation of hydrogen, the process of water splitting, and the functioning of metal-air batteries. Over the past ten years, researchers have explored the potential of non-precious electrocatalysts that utilize transition metals such as nickel, cobalt, and copper, as well as their respective oxides, for the purpose of water-splitting. This has been documented in various studies^41–44^. One of the possible candidates is lead (Pb), which is a widely available and low-cost metal with high electrical conductivity and chemical stability^44^. Pb has been used as a catalyst for various electrochemical reactions, such as carbon dioxide reduction, oxygen evolution, and organic oxidation^45,46^. However, its application for EHE has been relatively less explored. Pb-MOF for electrocatalytic hydrogen evolution is a novel material that has attracted attention for its potential application in clean energy production. Pb-MOF is a metal–organic framework composed of lead (Pb) metal centers and organic ligands that form a porous crystalline structure. Pb-MOF can act as an efficient electrocatalyst for the hydrogen evolution reaction (HER), which is the process of splitting water into hydrogen and oxygen using electricity. Pb-MOF has several advantages over conventional HER catalysts, such as high surface area, high activity, low cost, and tunable properties. Pb-MOF can also be modified by doping with other metals or heteroatoms to enhance its conductivity, stability, and catalytic performance. By incorporating different ligands and metal components into the MOF structure, the catalytic activity and stability of lead-based MOFs can be enhanced and tailored for different HER conditions. Pb-MOF for electrocatalytic hydrogen evolution is a promising material that could pave the way for the development of sustainable hydrogen economy. Herein, we prepared a novel Pb-MOF composite using the ultrasonic assisted method. Then the modified composite is used for water splitting application by electrochemical approaches.

## Experimental

### Synthesis of Schiff base ligand (H_2_L) linker

**Figure 1:**
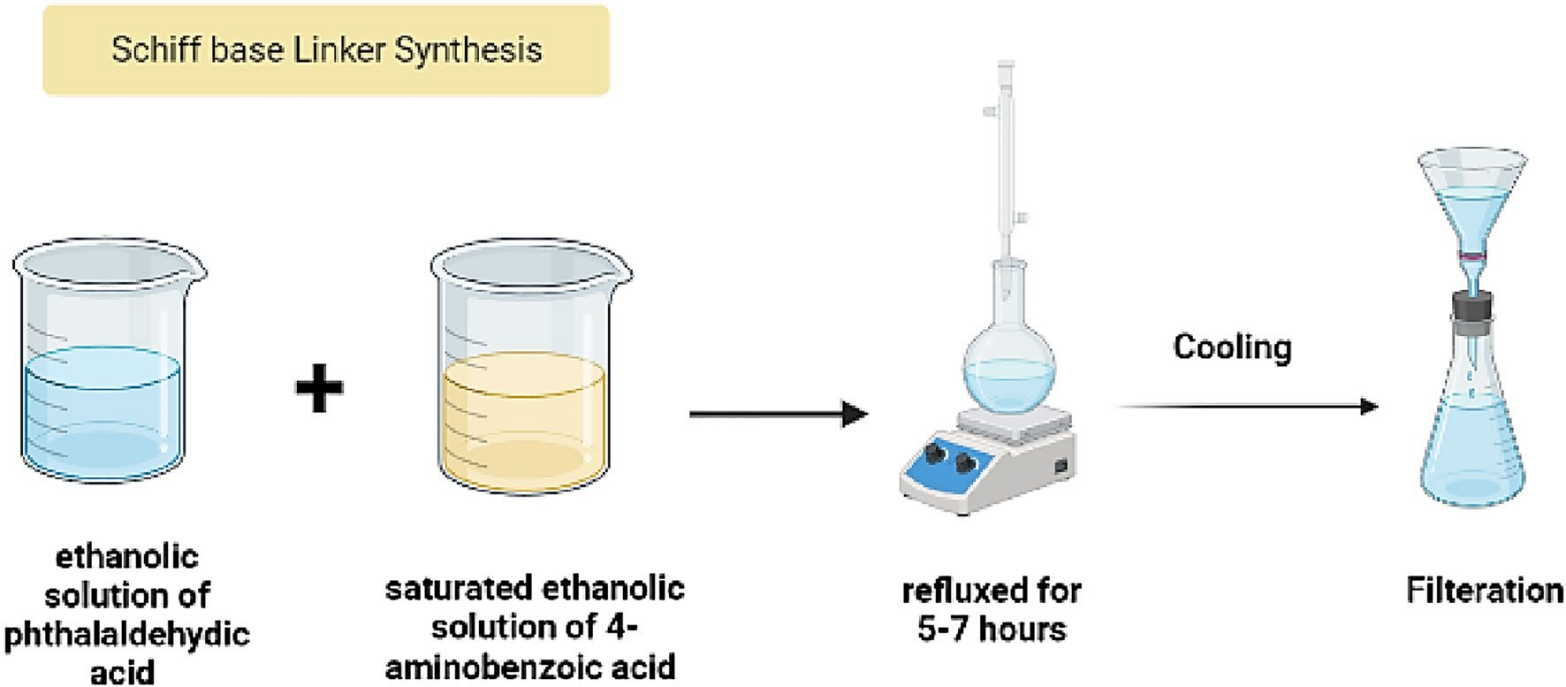
Preparation of H_2_L Schiff base ligand.

By combining a saturated ethanolic solution of phthalaldehydic acid (5 g) with another saturated ethanolic solution, the previously prepared Schiff base ligand (H2L) was created (see Fig. 1).

By a ratio of 1:1, of 4-aminobenzoic acid (5.47 g). The amine was mixed with the aldehydic solution before being allowed to reflux for 5–7 h. The resulting ligand was filtered, and the filtrate was repeatedly washed with cold ethanol until it was transparent. The solid ligand was dried over anhydrous calcium chloride in a desiccator. Figure 1 illustrates that the yield rate was 87 percent.

### Ultrasonic synthesis of Pb-MOF

**Figure 2:**
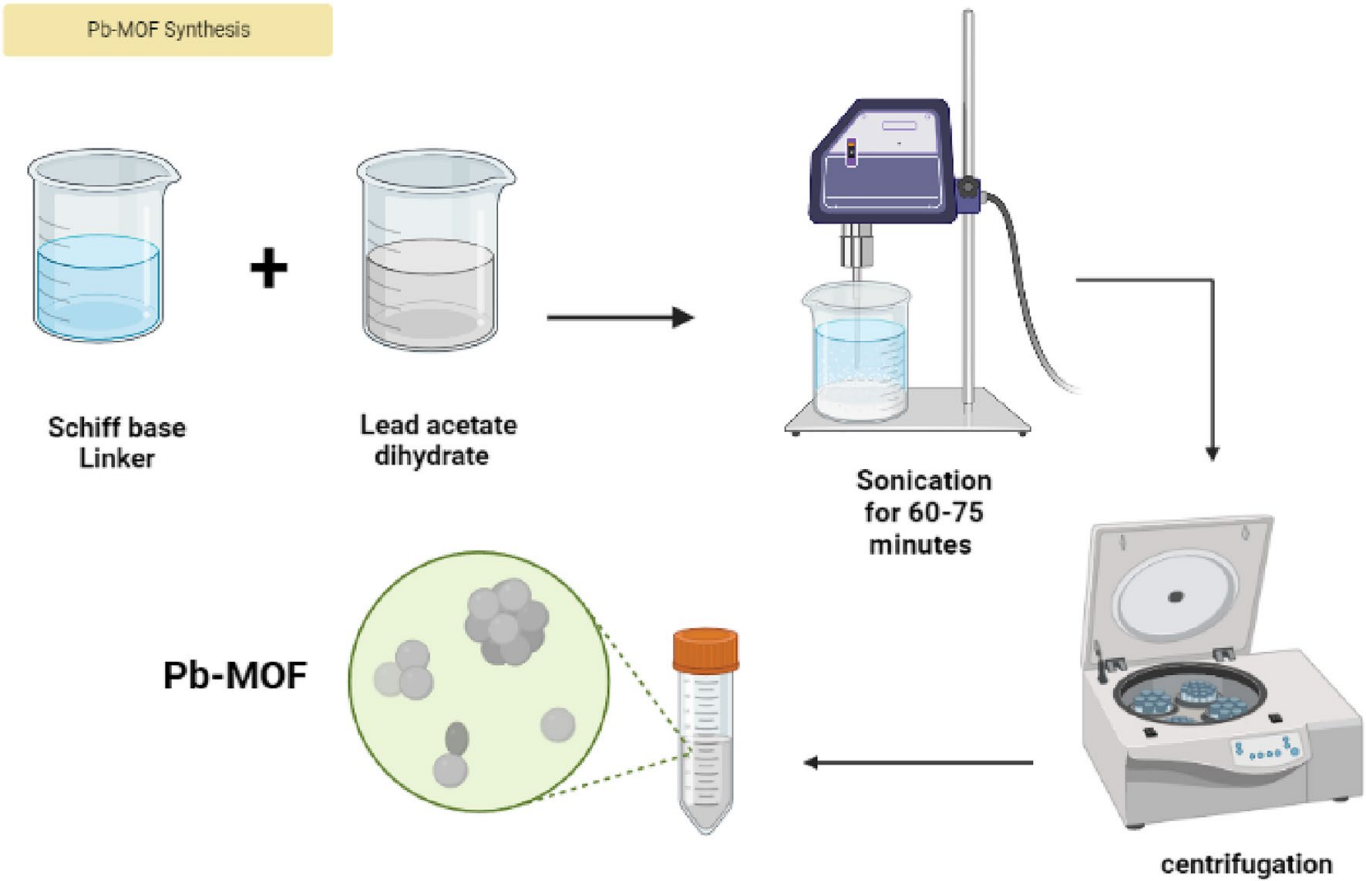
Preparation of Pb-based MOF.

The Schiff base ligand, denoted as H2L and having a molar quantity of 1 g and 3.7 mmol, was solubilized in 50 mL of absolute ethanol. A solution of lead acetate dihydrate (0.7 g, 1.85 mmol) was prepared by dissolving it in 30 mL of ethanol. The molar ratio of Lead acetate dihydrate to H2L is 1:2. The amalgamation of the two solutions was conducted, followed by their placement in a receptacle that was submerged in a water bath. The mixture was then subjected to sonication for a duration of 60–75 min, with a frequency of 40 kHz and alternating 1-s intervals of activation and deactivation. The experiment maintained a constant ultrasonic output of 60 watts. Following the required reaction time, the product was subjected to ultrasonic irradiation, after which it was isolated via centrifugation. The resulting precipitate underwent a thorough washing process using 50 mL of water and 10 mL of ethanol, which was repeated thrice. Finally, the precipitate dried at a temperature of 130 °C for a duration of 12 h. Subsequently, the product was cooled under ambient conditions at room temperature. Schematic representation of the preparation of Pb-MOF illustrated in Fig. 2.

## Results and discussion

### Characterization of Schiff base ligand (H_2_L)

The synthesis of a pre-existing ligand was conducted for the purpose of serving as an organic linker in the formation of metal–organic frameworks (MOFs)^47^. The ligand, denoted as H2L, was synthesized through the process of condensation between 4-aminobenzoic acid and 2-carboxybenzaldehyde. The facile methodology employed yielded a white-colored ligand, namely 2-(((4-carboxyphenyl)imino)methyl) benzoic acid. The elemental analysis results for carbon, hydrogen, and nitrogen were obtained, revealing percentages of 67.10% (calculated value = 66.91%), 5.36% (calculated value = 5.20%), and 4.20% (calculated value = 4.12%), respectively.

The outcomes were in concurrence with the computations derived from the prescribed equation (C_15_H_11_NO_4_), and the Schiff base ligand that was produced exhibited a distinct melting point of 270 °C, thereby validating its purity. As shown in Fig. S1, The IR spectrum analysis of the unbound ligand (H_2_L) revealed the absence of NH_2_ bands of 4-aminobenzoic acid and the emergence of a fresh v(CH=N) azomethine band at 1601 cm^−1^, as reported in reference^48^. The stretching bands of ν_asym_(COO–) and ν_sym_(COO–) were observed at 1468 cm^−1^ and 1321 cm^−1^, respectively, as reported in reference^21^. As shown in Fig. S2, The ligand's 1H-NMR spectrum exhibited a singlet signal at 5.8 ppm for HC=N with a single hydrogen atom, a singlet signal at 12.3 ppm for carboxylic protons with two hydrogen atoms, and multiple signals in the 6.5–7.9 ppm range that corresponded to the ligand's aromatic protons. The mass spectrum of the Schiff base ligand under investigation was primarily characterized by molecular ion peaks of moderate to somewhat high intensity. As per the findings of elemental investigations, it was observed that the mass spectrum of the Schiff base ligand exhibited a distinct parent peak at m/z = 269.07 amu, which was in agreement with the ligand moiety C_15_H_11_NO_4_ having an atomic mass of 269.25 amu. The peaks with values of 65, 74, 77.01, 80.99, 90, 105, and 133.04 amu can be attributed to distinct segments of the Schiff base ligand. The synthesized Schiff base ligand exhibited thermal stability and possessed a set of atoms that acted as donors of nitrogen and oxygen. Additionally, the ligand was soluble in solvents such as ethanol, DMF, and DMSO, and appeared white in color.

### Characterization of Pb-MOF

#### FT-IR analysis

**Figure 3:**
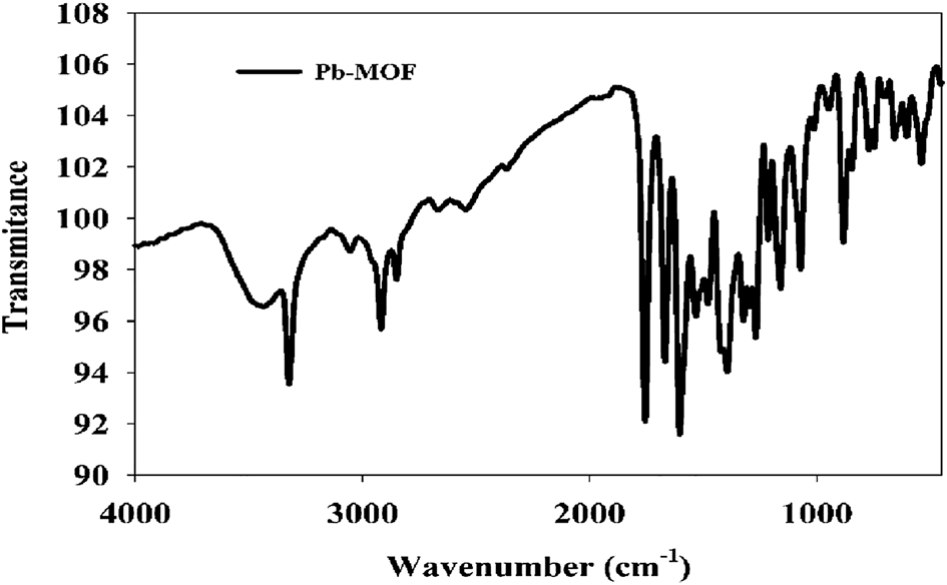
IR Spectra of Pb-MOF.

The absorption band with high intensity observed within the 3435–2918 cm^−1^ range is attributed to the O–H vibrations of water molecules that are present in the crystal structure, as reported in reference^4^. As shown in Fig. 3, The Fourier Transform Infrared (FTIR) spectrum of the Pb-MOF exhibited the emergence of robust bands at 1394 and 1602 cm^−1^, which can be attributed to the symmetric and asymmetric stretching modes of the coordinated (–COO) group, respectively. The observation suggests that the carboxyl group (–COOH) of H_2_L is involved in the coordination with lead. The carboxylate group exhibits bidentate chelating coordination in the Pb-MOF due to the fact that its Δv(COO) (Δν = νas(COO) − νs(COO)) is greater than that of the H2L ligand. The observed difference in antisymmetric and symmetric carbonyl stretching frequencies Δν for Pb-MOF was 247 (Δν = 1602 − 1394 = 208), which is significantly greater than that of H2L (Δν = 1468–1321 = 147). This indicates that the carboxylate moieties in the lead MOF coordinate in a chelating bidentate mode. A novel peak denoting the Pb–O bond was detected at 539 cm^−1^ in the metal organic framework under investigation^2^.

#### Powder X-ray diffraction pattern (PXRD)

**Figure 4:**
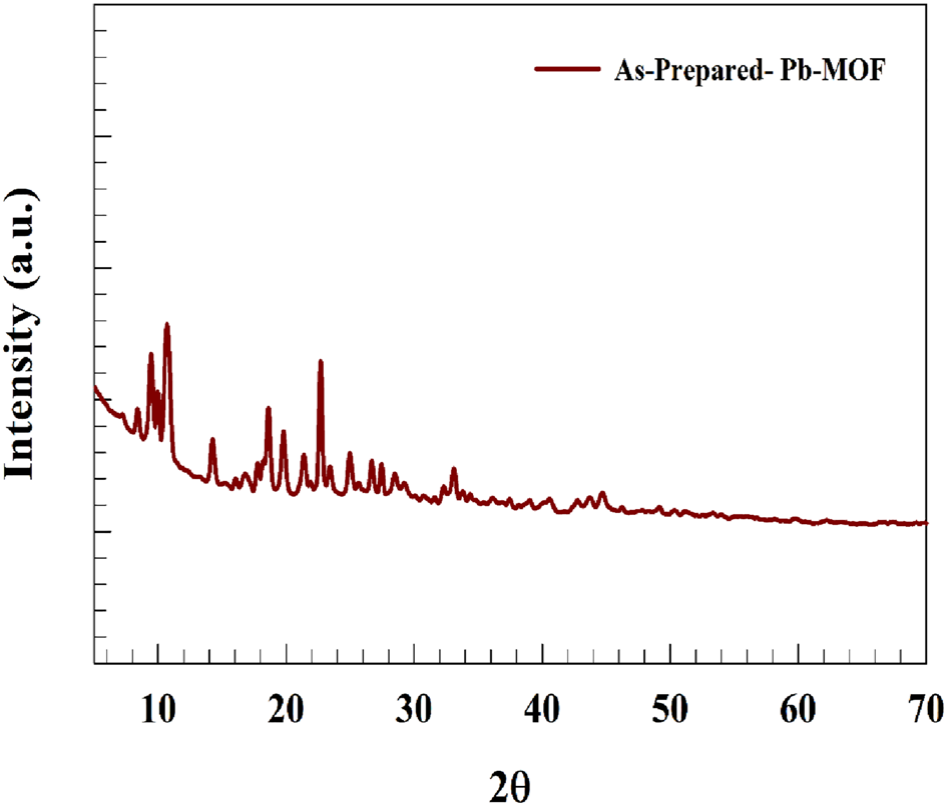
Powder X-ray diffraction patterns for (a) synthesized Pb-MOF,.

The X-ray diffraction (XRD) technique is highly valuable in furnishing data pertaining to the structure, average grain size, crystallinity, and other structural parameters. The X-ray powder diffraction pattern of the synthesized Pb-MOF exhibited a high degree of structural crystallinity, as depicted in the accompanying figure. The diffraction peaks observed in the Pb-MOF material are indexed at 9.9°, 10.6°, 10.8°, 14.2°, 18.6°, 19.8°, 22.6°, 26.6°, and 27.4°.(Fig. 4) The X-ray diffraction (XRD) pattern of the Pb-based metal–organic framework (MOF) obtained in this study is consistent with the findings reported in prior research^49,50^. Additionally, chemical structure of the Pb-MOF after stability test for 5 h of gas production. Thus, chemical structure changed that the PbO observed to generate instead of Pb-MOF. Severn characteristic peaks observed at 2θ equal to 18°, 29°, 31°, 36°, 49°, 54°. 60° according to reference card JCPDS card 01-078-1665^51,52^ (see Fig. S3). However, MOF structure changed to corresponding oxides with higher oxidation states. Whereas, various oxidation states of lead enhance the ability of the materials toward water splitting application. the conversion of the hybrid materials or organometallic materials through the catalysis process were extensively studied in literature to find out explanations for structure stability^53^. The chemical structure of the prepared Pb-MOF was finally estimated as represented in Fig. S4.

#### BET

**Figure 5:**
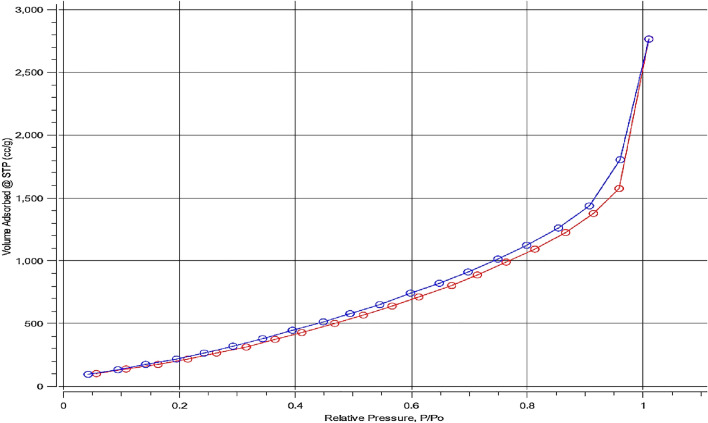
Adsorption–desorption isotherm for synthesized Pb-MOF.

The synthetic Pb-MOF's surface area and porosity were measured volumetrically using N_2_ adsorption. Standard N_2_ adsorption–desorption tests were performed at 77 K in order to look into the surface area, pore volume, and pore structure of Pb-MOF, as shown in Fig. 5. A type IV isotherm, which is typical of mesoporous materials, was seen in Pb-MOF. It was determined that the BET surface area was 1304.27 m^2^ g^1^. With an average pore size of 4.61 nm, the total volume of pores was calculated to be 2.13 cm^3^ g^1^.

#### SEM image of Pb-MOF

**Figure 6:**
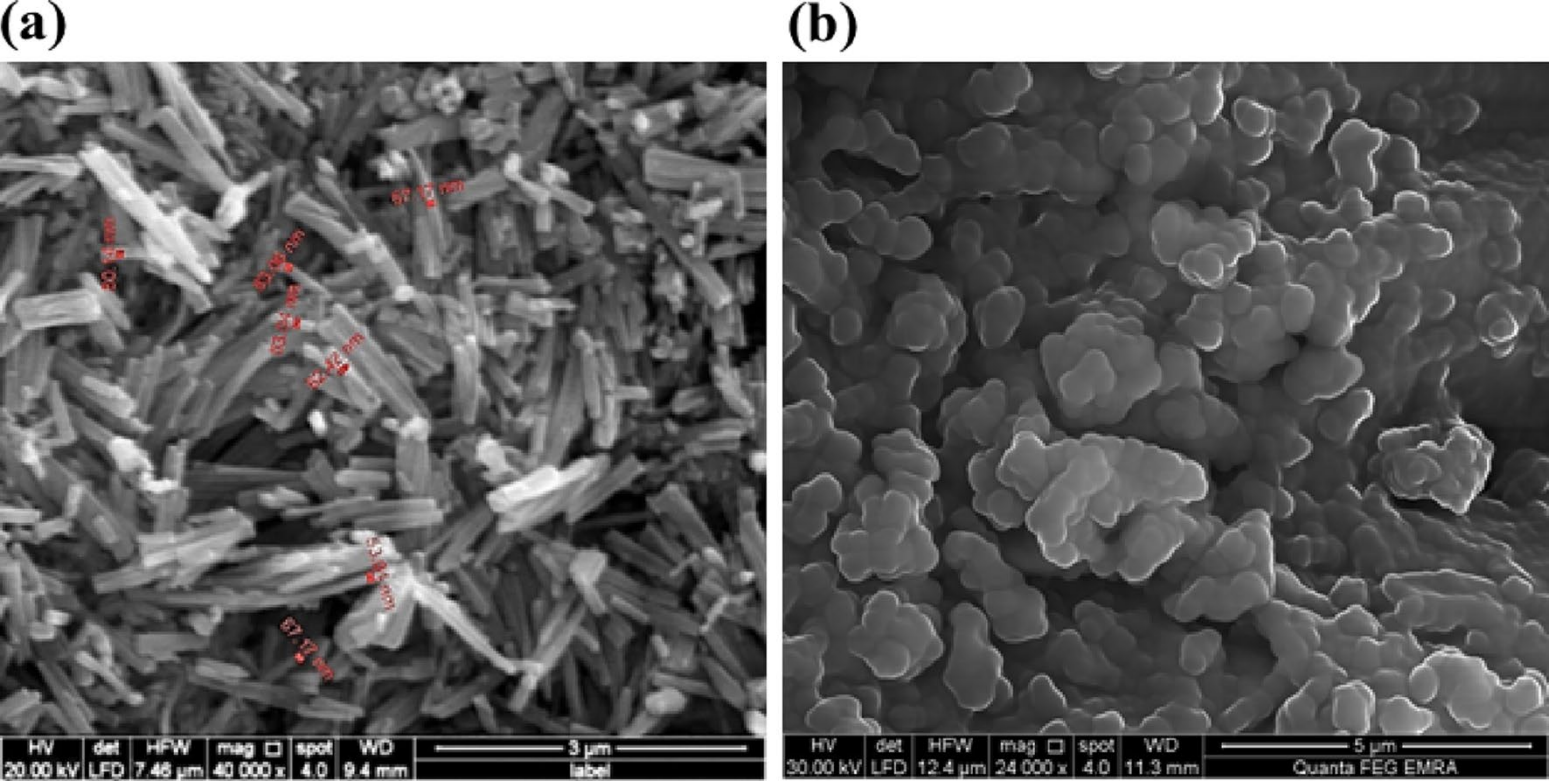
SEM images of synthesized (**a**) Pb-MOF, (**b**) Ligand.

The appearance, size, and structure of the sonochemically produced Pb-MOF were examined using scanning electron microscopy (SEM). It demonstrates that the particle size produced by ultrasonic irradiation is less than that produced by the solvothermal synthesis approach^3^. SEM images of the created Pb-MOF are shown in Fig. 6a. The results demonstrated that rod-shaped nanoparticles in the 53–83 nm range were successfully produced. Furthermore, the confirmation of Pb-MOF preparation was confirmed by comparing with unmodified ligand (see Fig. 6b).

#### EDX analysis

**Figure 7:**
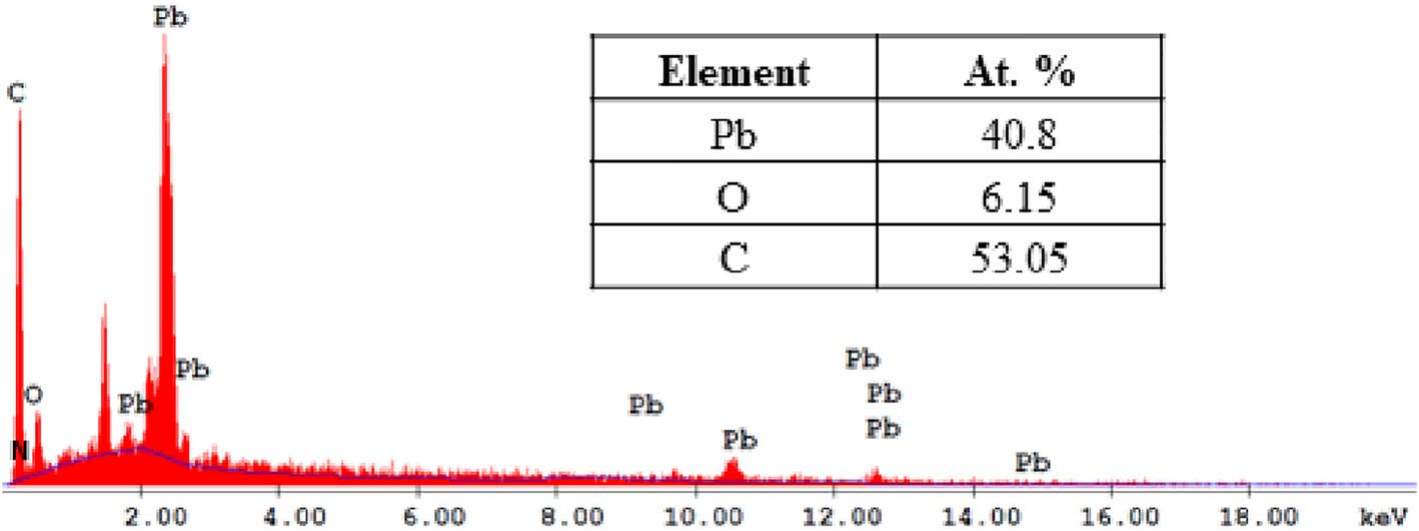
EDX spectrum of Pb-MOF.

The analysis of energy-dispersive X-ray spectroscopy (EDX) is a crucial technique that enables the determination of the elemental composition of a given sample. Furthermore, it is utilized to cartographically represent the horizontal dispersion of chemical constituents within the imaged region. The EDX spectrum of the produced Pb-MOF Fig. 7 confirmed the existence of lead (Pb), oxygen (O), and carbon (C). The percentage composition of the components present in the Pb-MOF was determined to follow the order of C>Pb>O. The identification of elements in the EDX spectrum suggests successful synthesis of the Pb-based MOF, with these elements potentially serving as active sites on the surface of the resulting adsorbents.

#### Thermal analysis

**Figure 8:**
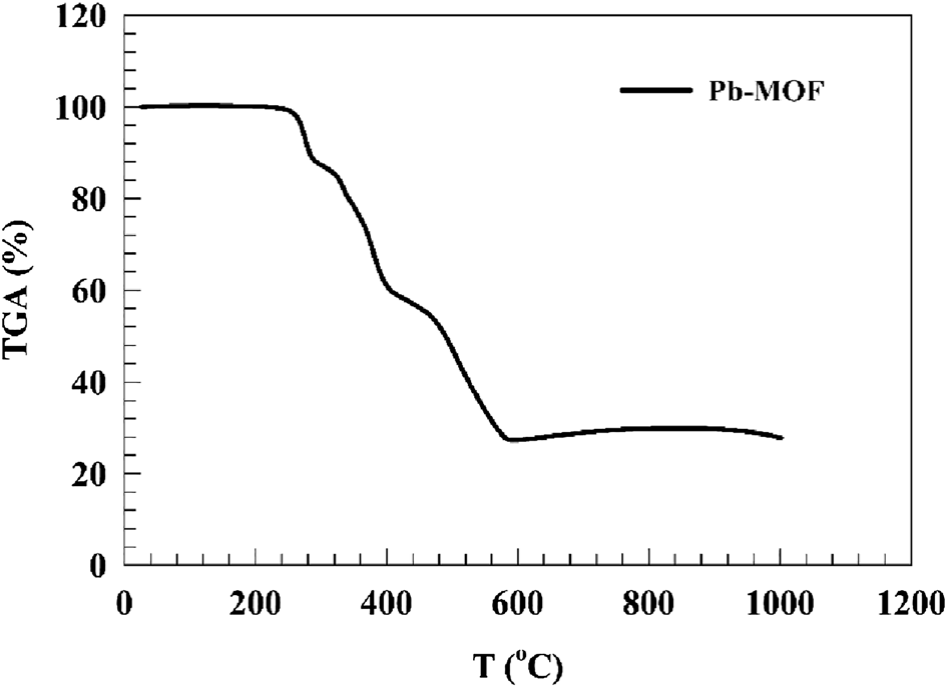
TGA of the Pb-MOF sample.

The process of thermal degradation of the composite material consisting of lead metal–organic framework (MOF) was investigated through the use of thermogravimetric analysis (TGA) (see Fig. 8). The compound exhibited three significant thermal transitions. During the initial stage, approximately 13.146% of weight loss can be attributed to the elimination of residual water molecules and ethanol solvent within the pore along with removal of residual unreacted carbon materials. This occurs through volatilization at temperatures ranging from 25 to 290 °C. The subsequent phase involves a sustained reduction in mass of approximately 8.192% within the temperature range of 290–340 °C. This step involves the elimination of coordinated water molecules from the Pb-MOF, resulting in the formation of novel active sites. During the third step, it was observed that the Pb-MOF sample experienced a reduction in weight of approximately 54.3% within the temperature range of 340.95–623.04 °C.

### Study of water splitting for lead-MOF

**Figure 9:**
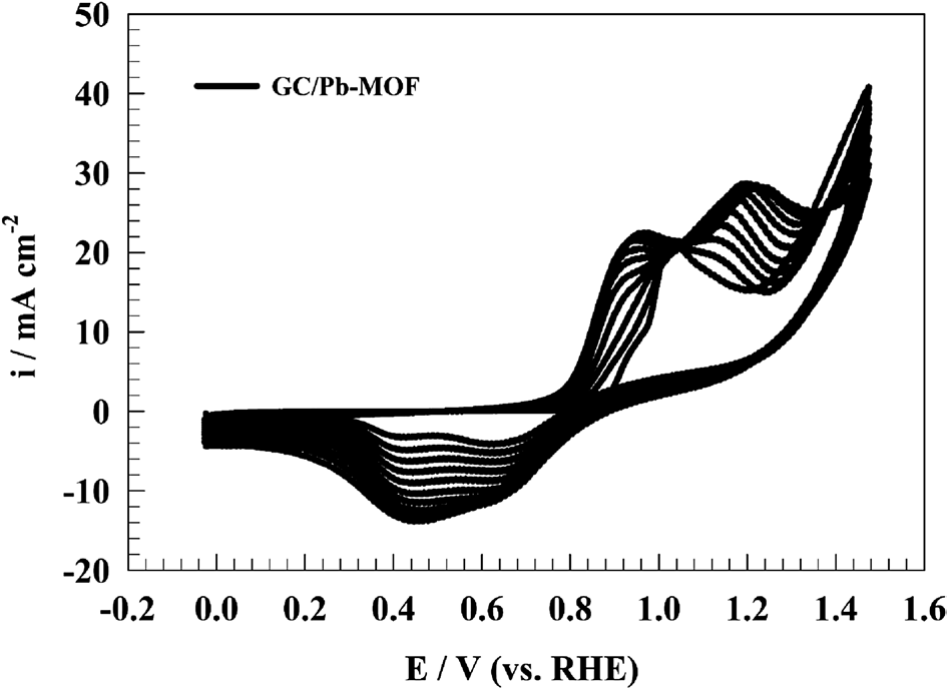
CVs of modified GC/Pb-MOF electrode in 1.0 M NaOH.

The electrochemical activity of the modified electrode was investigated in alkaline medium (1.0 M NaOH) by using cyclic voltammetry technique. First, the modified electrode was activated in the solution to generate the electrochemical active species. Thus, activation step was performed in alkaline medium in potential range of 0 to 1.5 V (vs. RHE). As represented in Fig. 9, repeated 50 CVs of modified GC/Pb-MOF electrode at scan rate of 50 mV s^−1^ in solution of 1.0 M NaOH. Two redox peaks were observed; 1st oxidation peak at potential of 0.9 V attributed to conversion of Pb^2+^ to Pb^3+^ while the 2nd oxidation peak at potential of 1.1 V attributed to conversion of Pb^3+^ to Pb^4+^. Additionally, the reduction peak observed at potentials of 0.42 and 0.7 V (vs. RHE) corresponding to the reduction of Pb^4+^ to Pb^3+^ and Pb^3+^ to Pb^2+^ respectively.

The process of oxygen evolution is of paramount importance in the conversion of chemical energy to electrical energy in fuel cells and batteries^54^. Various electrochemical methods have been utilized to determine the mechanism of the oxygen evolution reaction. One of the prevalent pathways for the electrochemical conversion of hydroxide to molecular oxygen involves a two-step electrochemical process. The initial step involves the adsorption of hydroxide ions onto the electrode surface, leading to the formation of OH_ads_ species. Subsequently, the adsorbed hydroxide group interacts with hydroxide ions present in the surrounding medium, resulting in the production of O_ads_. The ultimate stage involves the release of adsorbed atomic oxygen, resulting in the production of molecular oxygen. The operational framework of oxygen evolution reaction (OER) was established in the follows^42^:

1 $$ {\text{Pb}} + {\text{O}}H^{ - } \leftrightarrow {\text{PbOH}} + e^{ - } $$

2 $$ {\text{PbH}} + {\text{O}}H^{ - } \leftrightarrow {\text{Pb}}O^{ - } + {\text{H}}_{2} {\text{O}} $$

3 $$ {\text{Pb}}O^{ - } \leftrightarrow {\text{PbO}} + e^{ - } $$

4 $$ 2{\text{PbO}} \leftrightarrow 2{\text{Pb}} + {\text{O}}_{2} + e^{ - } $$

**Figure 10:**
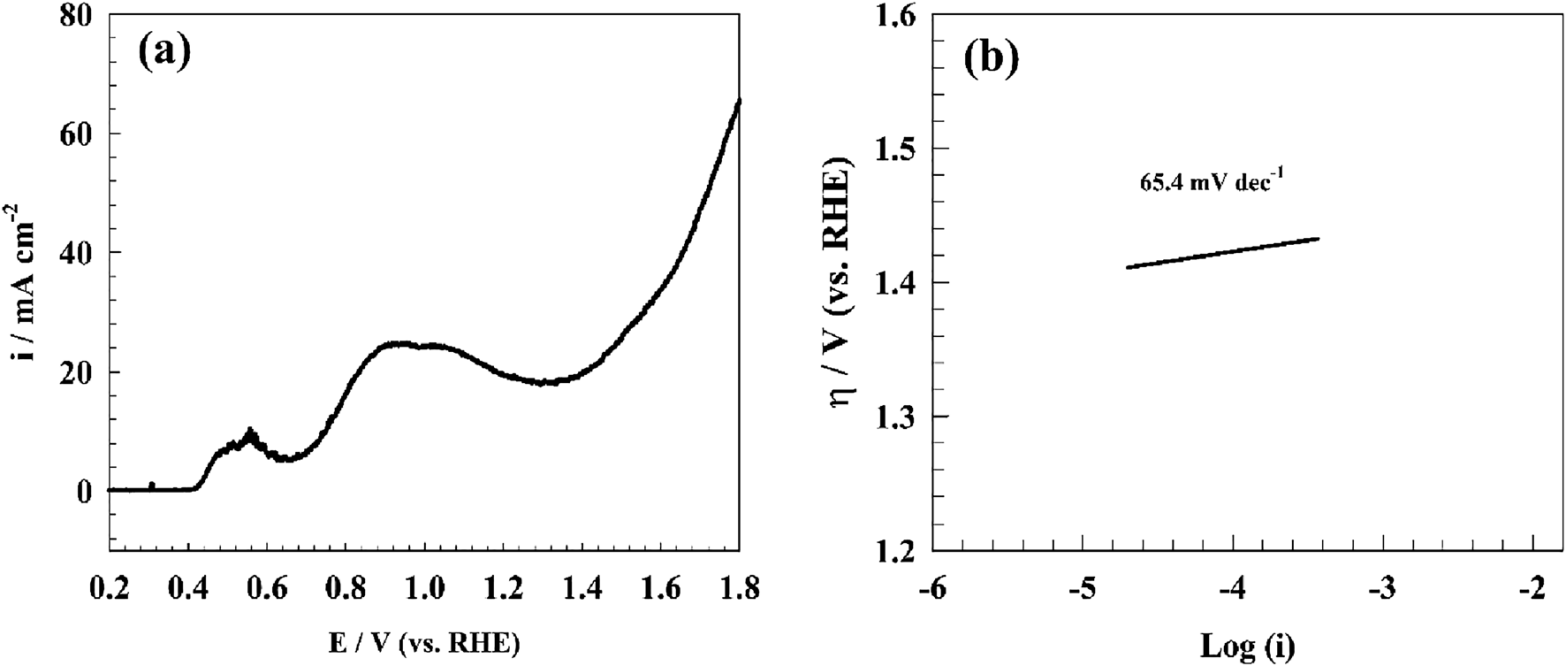
LSV of GC/Pb-MOF in 1.0 M NaOH for oxygen evolution, (**b**) Tafel Plot of OER over Pb-MOF.

Figure 10a illustrates the Oxygen evolution reactions (OER) observed on the GC/Lead Metal–Organic Framework (GC/Pb-MOF) under the influence of a 1.0 Molar concentration of Sodium Hydroxide (NaOH). As per the findings, a singular oxidation peak can be attributed to Pb^2+^ and Pb^4+^ ions, occurring at a potential of 0.5 and 1.05 V (vs. RHE) respectively^55,56^. The observed current density for OER in the Pb-MOF sample was found to be high. The modified electrodes exhibited a notable increase in current density, with the current peak attaining a value of 50 mA cm^−2^ at a potential of 1.7 V (vs. RHE) for GC/Pb-MOF. Figure 10b depicts a Tafel plot of the GC/Pb-MOF electrode for oxygen evolution reaction. The Tafel slopes pertaining to distinct modified surfaces have been determined, with a value of 64.4 mV dec^−1^ being obtained for GC/Pb-MOF. The Tafel slope for GC/Pb-MOF found to be comparable with other modified surfaces for OER in alkaline medium like NiCo nanosheets (41 mV dec^−1^)^57^, GC/LiCoO_2_ (48 mV dec^−1^)^58^, and GC/NiFe_2_O_4_ (98 mV dec^−1^)^59^ respectively.

**Figure 11:**
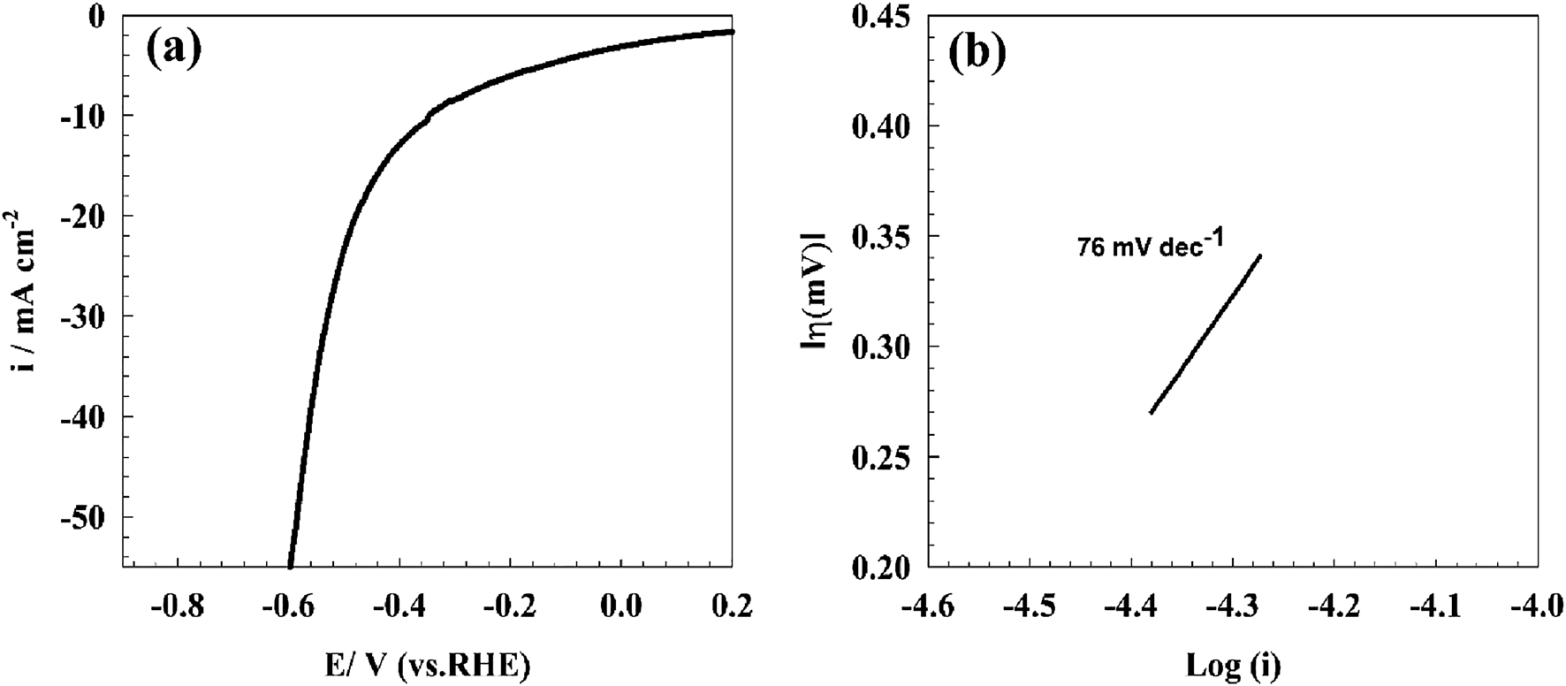
(**a**) LSV of GC/Pb-MOF in 1.0 M NaOH for hydrogen evolution reaction, (**b**) Tafel plot of modified GC/Pb-MOF.

The study focused on the investigation of hydrogen evolution reactions on a surface that has been modified by GC/Pb-MOF. The modified electrode's linear sweep voltammetry in a solution of 1.0 M NaOH is depicted in Fig. 11a.

The Pb-MOF that underwent modification exhibited a notable increase in current density, which can be attributed to the incorporation of organic molecules into the electrocatalyst frameworks. This modification resulted in an enhancement of both electronic and adsorption properties.

The subsequent mathematical expression has the capability to yield the hydrogen evolution reaction (HER) in an extremely basic environment^60,61^:

5 $$ {2}\;{\text{H}}_{{2}} {\text{O}} + 2e^{ - } \leftrightarrow 2\,\,H_{ads} + 2\,\,OH^{ - } \;\;\;\;\;{\text{Volmer}}\;{\text{Step-water}}\;{\text{dissociation}} $$

6 $${2\,\,H}_{ads}\leftrightarrow {H}_{2}\quad \mathrm{Tafel \,\,step}$$

7 $${\mathrm{H}2\mathrm{O}+ H}_{ads}+{e}^{-}\leftrightarrow {H}_{2}+\mathrm{O}{H}^{-}\quad  \mathrm{Heyrovsky \,\,step}$$

The initial stage of the hydrogen evolution reaction (HER) entails the adsorption of hydrogen ions (also known as the Volmer step) on the electrode's surface. Subsequently, the subsequent stage involves the amalgamation of two hydrogen ions that are adsorbed on the surface, which is commonly referred to as the Tafel step. Alternatively, it may involve the direct bonding between a hydrated proton present in the medium and an adsorbed hydrogen atom on the surface, which is known as the Heyrovsky step.

The determination of whether the first or second step is the rate-determining step for hydrogen evolution reactions can be approximated through the utilization of the Tafel polarization curve in the context of linear sweep voltammetry. Figure 11b depicts a Tafel plot of the GC/Pb-MOF electrode for hydrogen evolution reactions. The Tafel slopes pertaining to distinct modified surfaces have been determined, with a value of 76 mV dec^−1^ being obtained for GC/Pb-MOF. The provided Tafel slope value for GC/Pb-MOF matched with other reported for modified surfaces like Ni_2_Fe/N-doped porous C(83 mV dec^−1^)^62^, NiFe-LDH/MXene/Ni foam(70 mVdec^−1^)^63^, and Ni/NiO core/shell nanosheets(43 mV dec^−1^)^64^.

**Figure 12:**
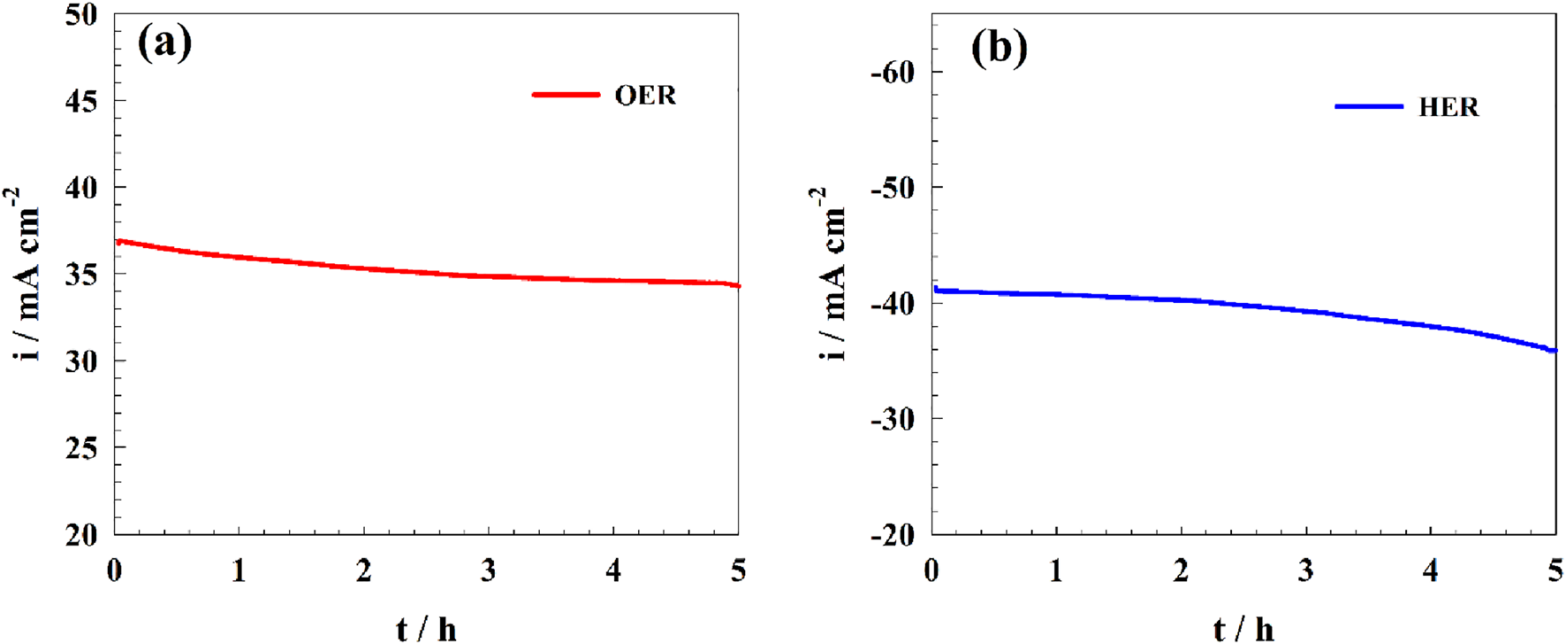
Chronoamperogram of the modified GC/Pb-MOF for OER and HER in alkaline medium.

Else, stability of the modified electrode toward gas production (i.e., OER, and HER) was investigated in alkaline medium using constant potential chronoamperometry. As represented in Fig. 12a, the durability of surface for oxygen evolution in 1.0 M KOH were tested for 5 h at potential of 1.7 V (RHE). The current decreased by 6.5% after 5 h. However, the stability of the modified electrode toward hydrogen production process was performed at potential of − 0.6 V (vs. RHE). Thus, the current of the electrode decreased by 13.8% of the initial values (see Fig. 12b).

**Figure 13:**
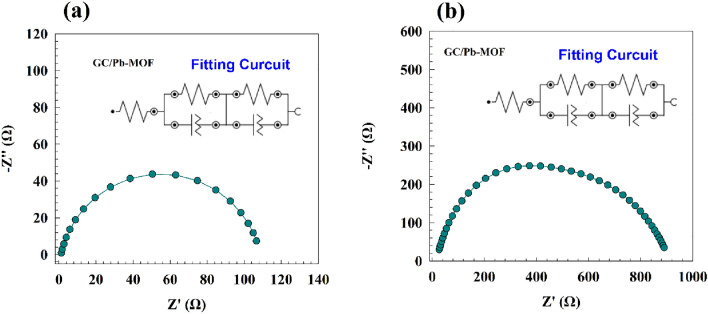
Nyquist plot of GC/Pb-MOF for (**a**) OER, and (**b**) HER.

The utilization of electrochemical impedance spectroscopy (EIS) was implemented in order to investigate the hydrogen and oxygen evolution phenomena in relation to the modified GC/Pb-MOF electrodes. The Nyquist plot for the GC/Pb-MOF modified electrodes in a 1.0 M NaOH solution at an AC potential of 1.6 V (vs. RHE) is depicted in Fig. 13a. The sample of MOF-Lead exhibited a semi-circular response in relation to the oxygen evolution reaction. The Nyquist plot's semi-circle is indicative of the charge transfer process. The EIS data pertaining to the process of oxygen evolution was subjected to fitting procedures utilizing NOVA software. The modified electrode's fitting circuit is represented by two resistance components that pertain to the solution resistance (Rs) and charge transfer resistance (Rc). The relationship between the charge transfer resistance (Rc) and the constant phase element (CPE) is established. The surface roughness of the Pb-MOF layers can be represented by the constant phase element. The resistances associated with charge transfer for electrodes that have been modified with GC/Pb-MOF are 106 Ω. The utilization of HER by EIS was demonstrated in Fig. 13b, where a constant AC potential of − 0.6 V vs. Ag/AgCl was employed. The Nyquist plot obtained from the modified electrode GC/Pb-MOF exhibited similar semi-circular plots, albeit with varying resistance magnitudes. The electrochemical production process may be regarded as a purely charge transfer phenomenon, as exemplified in the electrochemical impedance spectroscopy (EIS) data. The EIS outcome was subjected to fitting utilizing NOVA software. The GC/Pb-MOF electrode manifested a dual circuit configuration comprising a single cell linked to the solution resistance. The said cell consisted of a parallel connection between a resistance and the CPE (see Fig. 13 inset). Furthermore, the presence of a constant phase element suggests the existence of surface heterogeneity, which has an impact on the efficiency of hydrogen generation. The charge transfer resistance of the GC/Pb-MOF electrode is 570 Ω. High electrode activity is associated with a lower resistance value. The fitted parameters of EIS result were reported in Table. 1.

**Table 1:** Fitting parameters for OE and HER for corresponding modified GC/Pb-MOF electrode.

Electrode	Process	R_1_ (Ω)	R_2_ (Ω)	Q_1_		R_3_ (Ω)	Q_2_	
				Y_o_	n		Y_o_	m
GC/Pb-MOF	Oxygen evolution	12.911	26	3.9 $\times$ 10^–4^	0.7521	106	0.010049	0.627
GC/Pb-MOF	Hydrogen Evolution	15.55	127	2.45 $\times$ 10^–5^	0.8801	570	0.00017	0.679

## Conclusion

The present study reports the successful synthesis of a lead-based Metal–Organic Framework (MOF) utilizing the ultrasonic-assisted solvothermal technique. The synthesized materials underwent characterization to verify the establishment of the MOF structure. However, the Pb-MOF showed high activity toward water splitting application. The electrode reached the current density of 50 mA cm^−2^ at an overpotential of − 0.6 V (vs. RHE) for hydrogen evolution, and 50 mA cm^−2^ at potential of 1.7 V (vs. RHE) for oxygen evolution. The modified Pb-based electrode showed high durability toward water splitting applications. whereas the current of electrode decreased by 6.5, and 13.8% after 5 h of OER and HER respectively.

## Supplementary Information

Supplementary Information.

## Data Availability

The datasets used and/or analysed during the current study are available from the corresponding author on reasonable request.
