# Supplementary material for: Synthesis and characterization of lead-based metal–organic framework nano-needles for effective water splitting application
Source: Sci Rep. 2023 Aug 2;13:12531. doi: 10.1038/s41598-023-39697-z (PMC10397286; doi:10.1038/s41598-023-39697-z)
Supplement: Supplementary file 1 — Supplementary Information. [file 41598_2023_39697_MOESM1_ESM.docx]

# Title: Synthesis and Characterization of Lead-based metal-organic Framework nano-needles for effective water splitting application

**Ayman S. Eliwa, Mahmoud A. Hefnawy*^1^, Shymaa S. Medany, Reem G. Deghadi, Wafaa M. Hosny, Gehad G. Mohamed*^1,2^**

^1^ **Chemistry Department, Faculty of Science, Cairo University, Giza, Egypt.**

**^2^ Nanoscience Department, Basic and Applied Sciences Institute, Egypt-Japan University of Science and Technology, Alexandria, Egypt.**

- 1. **Materials and methods**

All used chemicals were of analytical grade and distillated water was used throughout all experiments. lead acetate dihydrate was supplied from Sigma-Aldrich and Phthalaldehydic acid (2-formylbenzoic acid) (purity 98%), 4-aminobenzoic acid (purity 98%) and lead acetate dihydrate (purity 98%) were purchased from Sigma-Aldrich. Also, the absolute ethyl alcohol was spectroscopic pure from BDH. De-ionized water was saved from all glass equipments and used in all preparations.

- 1. Instruments and measurements

The FT-IR spectra obtained using a Perkin-Elmer 1650 spectrometer (4000-400 cm^-1^) as KBr pellets were used in this study. TG and DTG were carried out from room temperature to 1000 °C using a Shimadzu TG-50H thermal analyzer at a rate of 5 ºC/min. A SEM Model Quanta 250 FEG (Field Emission Gun) linked to an EDX unit (Energy Dispersive X-ray Analyses) was used to capture an image of the MOF, with an accelerating voltage of 30 K.V., magnification of 14 up to 1000000, and resolution for Gun (National Research Center, Egypt). The X-ray diffraction (XRD) was recorded in Egypt Nanotechnology Center (EGNC) using a Bruker D8 Discover (Bruker AXS Inc., 35 KV, 30 mA) X-ray diffractometer with a step size of 0.02 and speed scan of 0.016 for 2h with 2 varying between 5 and 50.

Gas adsorption tests using N2 as the adsorptive gas at 77 K were used to determine the BET surface area and pore size distribution. Prior to the adsorption tests, the materials were evacuated for 4-12 hours under high vacuum. The Brunauer-Emmett-Teller (BET) theory was used to calculate the analysis, which was done with a Nova Touch LX2 analyzer. UV-Vis spectra were recorded using a Shimadzu UVmini-1240 UV-Vis spectrophotometer. Attension-Theta of Biolin Scientific assessed the contact angle at Egypt Nanotechnology Center, Cairo University, El-Sheikh Zayed, Egypt.

- 1. **FTIR for Ligand**


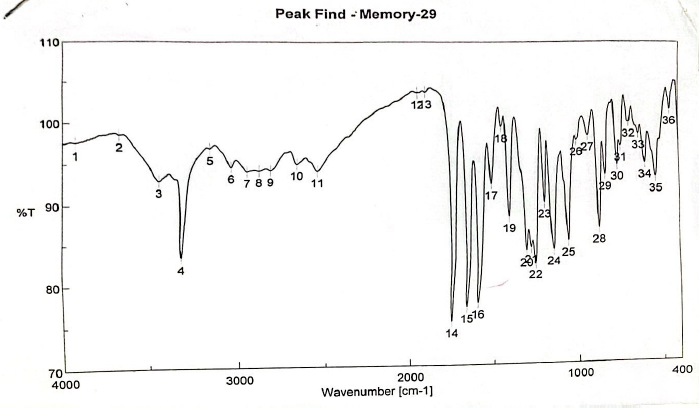


**Figure S1**: FTIR spectra for Ligand

**d. NMR for Ligand**


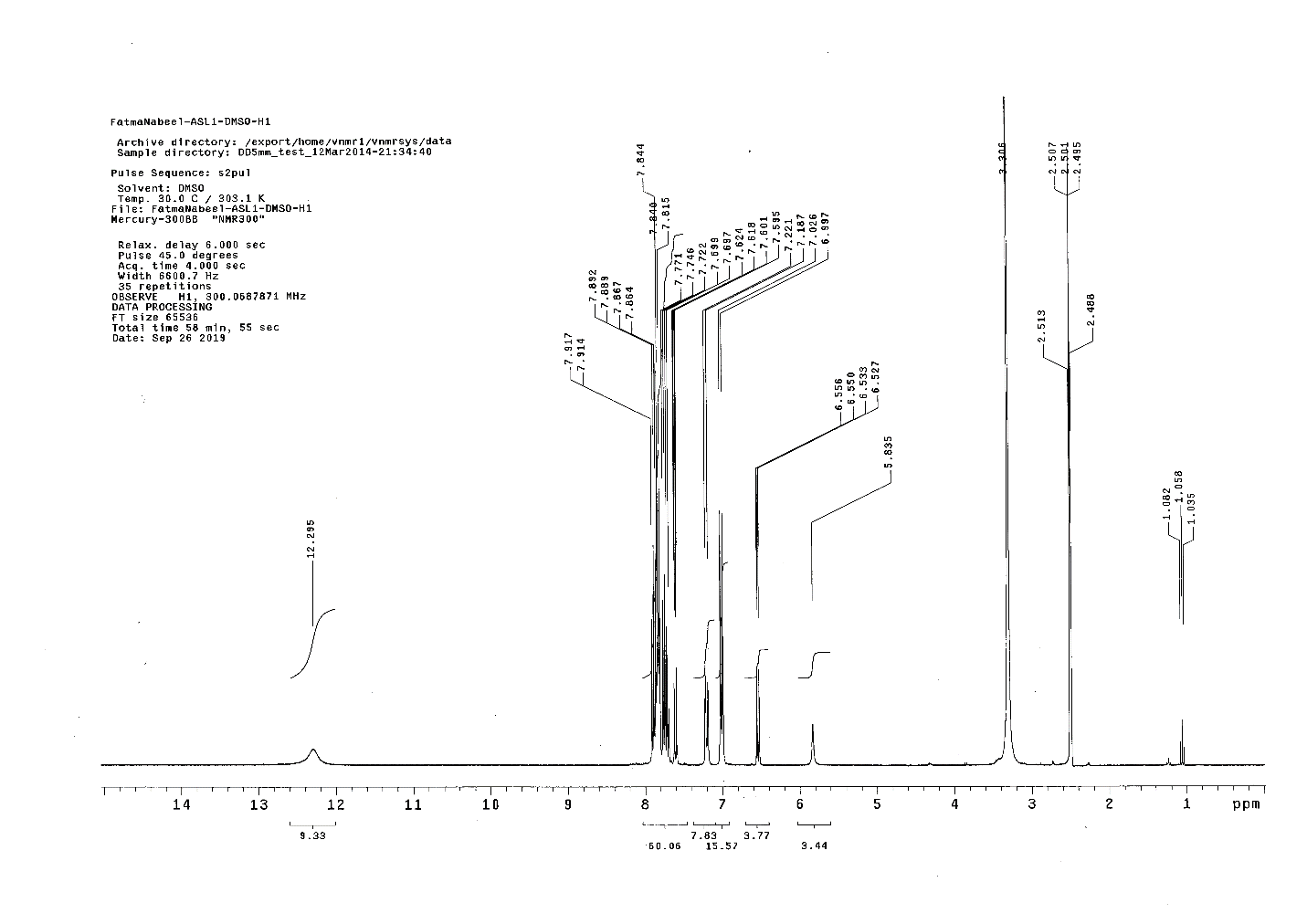


**Figure S2**: NMR for Ligand

**e- XRD for Pb-MOF after stability**


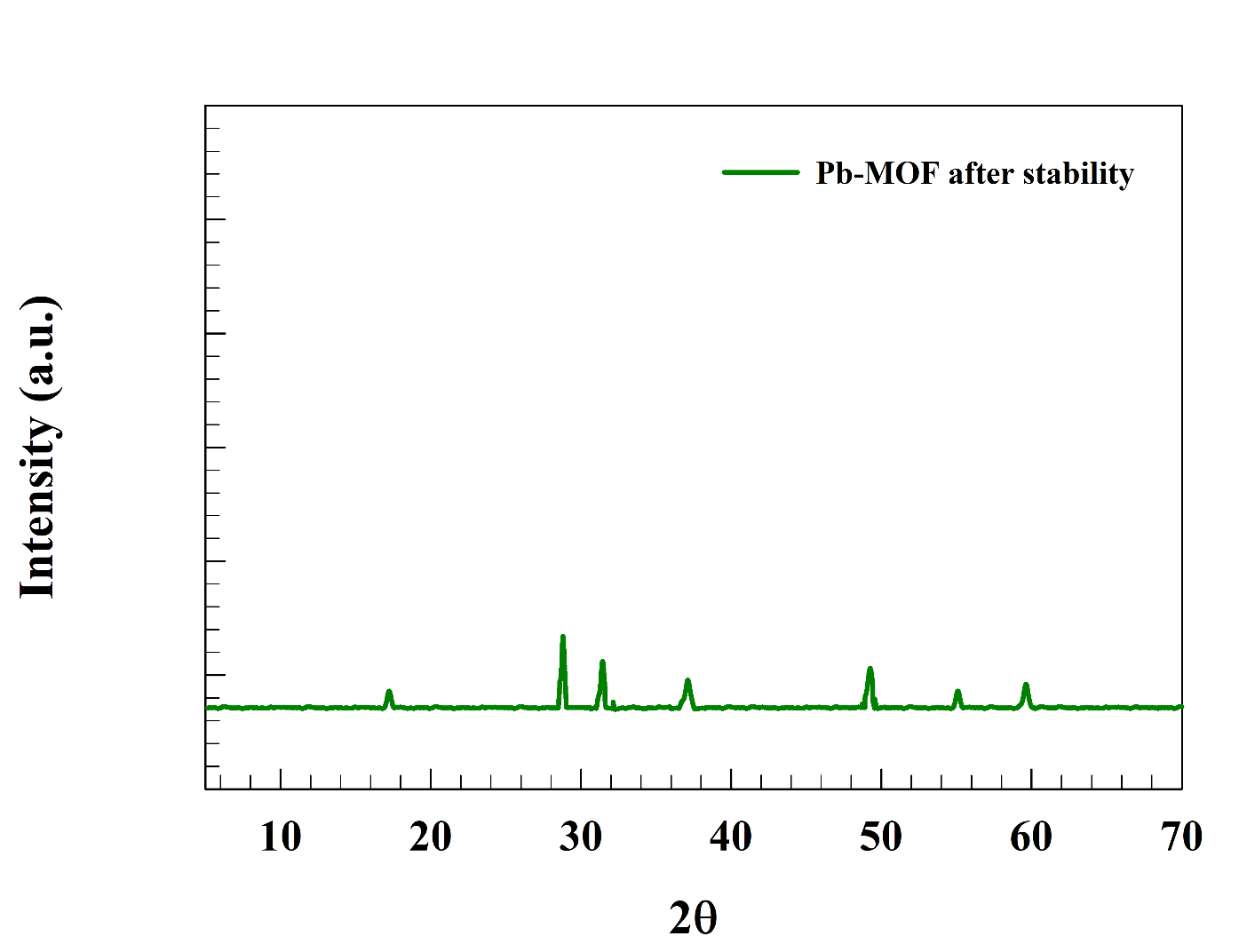


**Figure S3**: Pb-MOF after stability test

**f. Chemical structure of Pb-MOF**


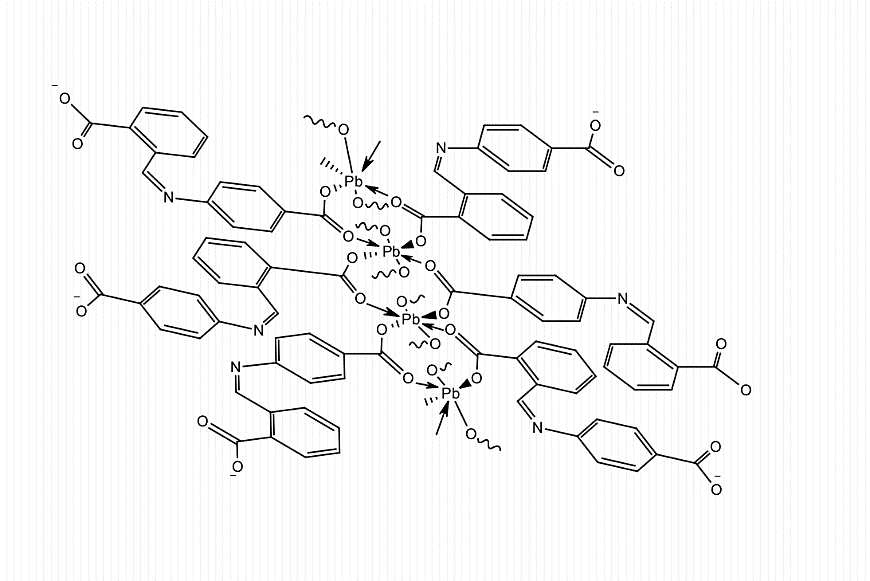


**Figure S4.** Structure of synthesized Pb-MOF
